# Critical role of empiric antitoxin therapy in suspected *Clostridium baratii* infection: a case report

**DOI:** 10.1128/asmcr.00153-25

**Published:** 2026-02-23

**Authors:** Maya Polashenski, Michael J. Perry, Abhimanyu Aggarwal

**Affiliations:** 1 Rochester General Hospital 6932, Rochester, New York, USA; 2 New York State Department of Health, Wadsworth Center 116287, Albany, New York, USA; Rush University Medical Center, Chicago, Illinois, USA

**Keywords:** botulism, descending paralysis, antitoxin

## Abstract

**Background:**

*Clostridium baratii* infections, though rare, can result in life-threatening botulism due to the production of botulinum neurotoxin. The rapid administration of antitoxin is the cornerstone of treatment to mitigate neurological damage and prevent mortality. Given the difficulty in obtaining rapid diagnostic confirmation, empiric administration of antitoxin is often justified.

**Case Summary:**

This paper describes a case of *C. baratii* infection with complete recovery after empiric treatment with antitoxin prior to confirmatory testing returning. Additionally, this manuscript explores the pathophysiology of *C. baratii*, the diagnostic challenges, and the rationale for early empiric antitoxin therapy.

**Conclusion:**

The nonspecific nature of early symptoms of botulism necessitates a high degree of clinical vigilance and preparedness. This case highlights the need for including botulism in the differential diagnosis for abnormal neuromuscular presentations and how rapid empiric therapy can be lifesaving.

## INTRODUCTION

Botulism is a severe paralytic disease caused by neurotoxins produced by *Clostridium spp*., most commonly *Clostridium botulinum*, but also *Clostridium baratii* and *Clostridium butyricum* (1). Among these, *C. baratii* has emerged as a sporadic pathogen in botulism cases, predominantly in infants but also in adults with underlying conditions or unique exposure scenarios. There are several modes of transmission of botulism, including ingestion of botulinum neurotoxin (BoNT) in food (foodborne botulism), inhalation of the neurotoxin (inhalation botulism), exposure to the neurotoxin from a colonized wound (wound botulism), and exposure related to injection of neurotoxin for cosmetic or therapeutic treatment (iatrogenic) (2). These are the types of botulism typically implicated in outbreaks, especially foodborne transmission. There are also syndromes described when there is intestinal colonization of botulinum toxin-forming *Clostridium spp*., which can also cause botulism in the right patient population. This is typically seen in infants (infant botulism), but also adults (adult colonization botulism) (3).

Delayed treatment of botulism with antitoxin can result in irreversible neuromuscular damage or death. Due to the time-sensitive nature of the disease and the slow turnaround of confirmatory laboratory diagnostics, empiric antitoxin therapy plays a critical role. We present a case of confirmed *Clostridium baratii* botulism in which adult colonization was the suspected source of infection, and the patient made a full recovery after receiving empiric botulism antitoxin. The importance of immediate intervention cannot be overstated enough in suspected *C. baratii* infections, where clinical suspicion is often the primary driver of management.

## CASE PRESENTATION

A 69-year-old man with a medical history significant for hypertension, hyperlipidemia, and obstructive sleep apnea initially presented to the emergency room with diplopia and slurred speech. He had returned from a trip to Florida 2 days prior and was in his normal state of health when his symptoms began. Upon presentation to the hospital, he became obtunded due to acute hypercapnic and hypoxemic respiratory failure requiring urgent intubation and admission to the intensive care unit (ICU). Vital signs on hospital day 1 showed a normal heart rate, no fever, and resolution of hypoxemia with intubation. Pertinent initial neurologic examination included fixed pupils and ptosis bilaterally. There was no evidence of epileptiform activity on the electroencephalogram and no acute stroke on magnetic resonance imaging of the brain. Lumbar puncture showed no nucleated cells on fluid analysis, with negative cerebrospinal fluid Gram stain and culture and a negative meningoencephalitis polymerase chain reaction (PCR) panel. An autoimmune panel of the cerebrospinal fluid (CSF) was positive for anti-GM1 antibodies.

His neurological examination on day 2 of hospitalization was significant for progressive descending paralysis and areflexia, adding botulism, Guillain-Barré syndrome (GBS), and myasthenia gravis to the differential diagnosis. He remained ventilator-dependent due to respiratory muscle weakness, but his vital signs were otherwise stable. Further history revealed the patient had traveled with his son, who ate all the same food and had no symptoms. He did not have recent antibiotic use nor a history of immunosuppression or abdominal surgeries. At this point, the New York State Department of Health and Centers for Disease Control and Prevention (CDC) were contacted for the request of botulism antitoxin to be given empirically. Plasma exchange therapy was also initiated. On hospital day 3, the botulism antitoxin arrived, and the patient was promptly treated. He also underwent six sessions of plasmapheresis on hospital days 2, 6, 8, 10, 12, and 14 due to concern for an autoimmune process. On hospital day 2, he had a serum antigen test sent to the New York State Department of Health Laboratory for neurotoxin-producing strains of *Clostridium spp*., which was negative. Subsequently, on hospital day 7, a rectal swab and a stool sample were collected for botulism toxin testing and sent to the New York State Department of Health Laboratory, which returned positive for *Clostridium baratii* toxin F by PCR and culture. These results were available on hospital day 15.

**Fig 1:**
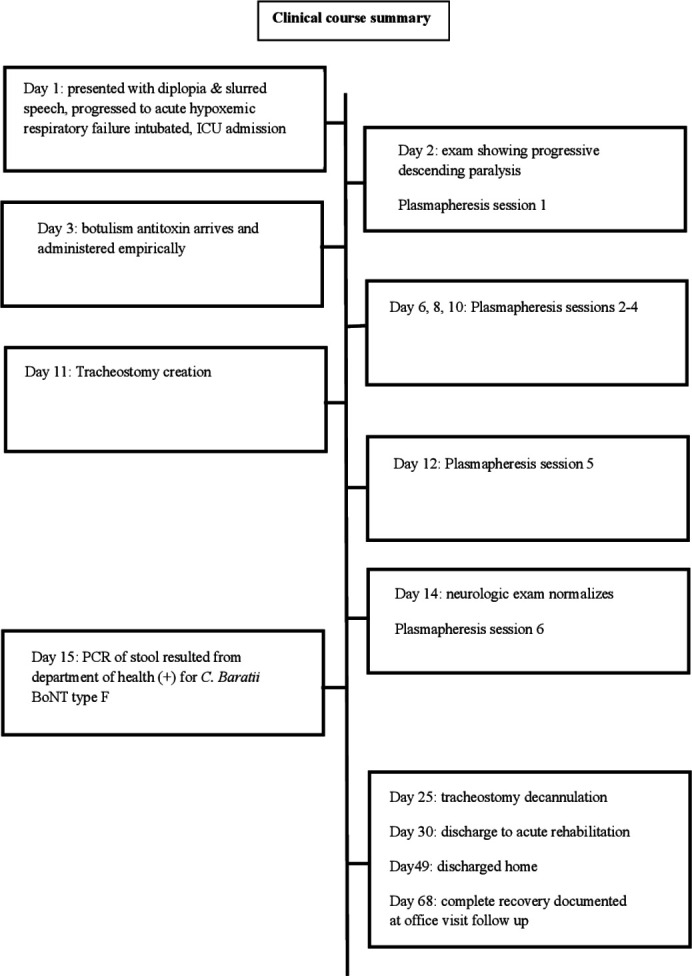
Timeline of clinical course summarized.

The patient showed clinical signs of improvement with regaining neurological function on hospital day 14, 11 days after receiving botulism antitoxin. He underwent tracheostomy creation on hospital day 11 but was successfully decannulated 2 weeks later during the same admission. After being hospitalized for 30 days, he was discharged to acute rehabilitation, where he stayed for 19 days and then was discharged home. He made a complete recovery by day 65 status post antitoxin administration, including ambulating independently, driving, and returning to work. According to follow-up visits, he has remained in good health for nearly 2 years after the event. His primary care physician documented that he was interviewed by the CDC following discharge, with no source of foodborne botulism identified or signs of an outbreak. His hospital course is summarized chronologically in Fig. 1.

## DISCUSSION

*C. baratii* is a gram-positive, spore-forming anaerobe capable of producing botulinum neurotoxins (BoNT). These toxins, particularly type F produced by *C. baratii*, interfere with acetylcholine release at the neuromuscular junction, leading to flaccid paralysis (4). While type F botulinum toxin tends to have a shorter duration of effect compared to other serotypes, it is still a dangerous toxin and requires urgent medical intervention.

According to the CDC, there were 273 cases of botulism reported in 2021, which is the most recent data listed on their website. Of those cases, 242 were confirmed in a laboratory, and 30 were probable. Further breakdown reveals that 181 cases were infant, 66 cases were wound, 22 cases were foodborne, and 4 cases were “other,” 1 of which was listed as adult colonization, also referred to as intestinal botulism, and is hypothesized to be like the mechanism of infant botulism. In this form of botulism, toxin-producing strains of *Clostridium spp*. colonize the gastrointestinal tract and produce botulinum toxin *in situ* (3). *C. baratii* can also produce BoNTs, particularly type F, which leads to botulism in humans. While it is more commonly associated with infant botulism, rare cases of intestinal botulism in adults have been reported (5).

Unlike other forms of botulism, where exposure to the toxin is via ingestion, injection, or inhalation, intestinal botulism involves bacterial spores germinating in the host’s intestines. Exposure to spores may be related to soil, dust, or improperly handled foods (3). Once released, the toxin disrupts the release of acetylcholine at neuromuscular junctions, resulting in flaccid paralysis (4). Adult cases typically occur in individuals with underlying risk factors, such as disrupted gut flora or structural abnormalities in the gastrointestinal tract, including recent antibiotic use or abdominal surgeries (3). The neurological signs are identical to other forms of botulism, including descending paralysis, diplopia, ptosis, dysphagia, and respiratory failure in severe cases. The diagnosis is made by identifying the botulism neurotoxin implicated in the stool, serum, or gastric fluid; for this case, it was BoNT type F. This is done by detecting the presence of toxins using an enzyme-linked immunosorbent assay or by detecting toxin genes using PCR. Additionally, stool, serum, or gastric fluid can be sent for culture. For this case, both culture and biochemical analyses identified the organism as *Clostridium baratii* at the State Department of Health Laboratory. This allowed us to successfully obtain, isolate, and further characterize the organism using both PCR and matrix-assisted desorption/ionization-time of flight mass spectrometry (MALDI-TOF MS) methods. PCR testing served as a screening assay to detect the presence of toxin-producing genes associated with *C. botulinum* serotypes A, B, E, F, and *Clostridium baratii* type F. In this case, the isolate tested negative for the toxin genes associated with serotypes A, B, E, and *C. botulinum* type F; however, the toxin gene corresponding to *Clostridium baratii* serotype F was detected. To further assess toxin production, the isolate was analyzed using the MALDI-TOF MS Endopep assay to determine whether active toxin was being produced (6). This testing confirmed the presence of active serotype F toxin, consistent with the PCR findings. Together, these results demonstrate that the isolate was *Clostridium baratii* producing active botulinum neurotoxin serotype F.

There are challenges in obtaining a diagnosis that are multifactorial. First, there is a delay in confirmatory diagnostic tests, since they need to be sent out to a public laboratory, and culture is often needed to verify active toxins that can be screened with biochemical testing. Second, due to the uncommonness of botulism, especially lack of awareness of strains beyond *Clostridium botulinum*, clinicians may overlook *C. baratii* as a potential pathogen in descending paralysis. Third, due to the overlap with presentations from other neuromuscular conditions, such as GBS and myasthenia gravis, there may be a delay in pinpointing the correct diagnosis. A list of differential diagnoses is outlined in Table 1. Specific to this case, GBS was considered, especially given positive serum anti-GM-1 antibodies in the patient’s laboratory investigations. The lumbar puncture fluid analysis did show a very mild elevation in CSF protein with normal cell counts, which can be seen in GBS; however, protein elevation is typically more drastic, owing to the albuminocytologic dissociation phenomenon. Although the presentation (mainly descending instead of ascending) paralysis and lack of preceding infectious history made GBS lower on the differential than botulism, the patient still received six sessions of plasmapheresis in addition to empiric antitoxin, further highlighting how close these two flaccid paralysis syndromes can present. Other infectious causes of neuromuscular weakness, including tick paralysis or arboviruses (7), are considered less likely with no tick found on physical exam and negative microbiological workup during hospitalization for West Nile Virus, given recent travel to Florida (Table 2).

**TABLE 1:** Description of differential diagnoses for botulism

Condition	Distinguishing features
Guillain-Barré syndrome (GBS)	Paralysis is typically ascending and often preceded by a recent infection (respiratory or gastrointestinal); sensory symptoms (paresthesias and pain) are common; deep tendon reflexes are absent early; CSF may show albuminocytologic dissociation (2)
Miller Fisher syndrome (a GBS variant)	Triad of ataxia, areflexia, and ophthalmoplegia; can initially mimic botulism due to prominent cranial nerve involvement; diagnosis supported by presence of anti-GQ1b antibodies (2)
Myasthenia gravis	Weakness is fluctuating and fatigable; reflexes are usually preserved; symptoms often respond dramatically to cholinesterase inhibitors; associated with anti-acetylcholine receptor antibodies (2)
Lambert-Eaton myasthenic syndrome (LEMS)	Often associated with an underlying malignancy; weakness may improve with initial effort/contraction; autonomic symptoms are common (2)
Brainstem stroke	Usually causes asymmetrical or focal deficits; may present with altered consciousness or other signs of central nervous system involvement; neuroimaging will show brainstem lesions (2)
Tick paralysis	Ascending flaccid paralysis that can mimic GBS; occurs after prolonged attachment of a toxin-producing tick; symptoms often resolve rapidly after tick removal; ticks that are implicated include *Dermacentor andersoni*, *Dermacentor variabilis*, or *Ixodes holocyclus* (8)
Arboviruses	There are reports of neuroinflammation causing symptoms of acute paralysis, ophthalmoplegia, and sometimes long-term CNS defects associated with Zika virus, West Nile virus, Usutu virus, Dengue virus, Chikungunya virus, and Japanese encephalitis virus; the reports describe the paralysis to be ascending weakness or triggering infection for GBS and often associated with meningitis or encephalitis (7)
Poisons/intoxications	History of exposure to toxins Organophosphates can cause neuropathy that can lead to “distal axonal degeneration and demyelination of the central and peripheral axons” (8), which can cause paralysis in severe cases Flaccid paralysis can be seen with ingestion of mollusks that are contaminated by certain toxins (dinoflagellate-produced saxitoxin or brevetoxin B), the proposed mechanism being from sodium channel blocking toxin that can lead to flaccid weakness of the extremities and death due to respiratory muscle weakness (8) There are toxins in Buckthorn fruits (endemic in the southwest United States and Mexico) and lead to a distal, symmetric, ascending flaccid paralysis of the limbs with a mixed demyelination, which can mimic GBS (8)

**TABLE 2:** Laboratory data

LP fluid analysis	Micro (serum)	Micro (CSF)	Other lab tests
RBC: 0 Nucleated cells: 0 Protein: 47 mg/dL Glucose: 101 mg/dL Appearance: clear Color: colorless	Blood cultures: negative Lyme antibody: negative West Nile Virus antibodies (IgG and IgM): negative Erlichia PCR: negative Babesiosis PCR: negative Anaplasma PCR: negative HIV 1 and 2 combo antigen/antibody EIA: negative	CSF Gram stain: no WBCs and no organisms seen. CSF culture: no growth Cryptococcal antigen: not detected Fungal culture: no fungus isolated Meningitis/encephalitis PCR panel: negative	MUSK antibodies: negative Ganglioside antibodies: GM-1: positive, all other serotypes negative (Asialo-GM1, GM2, GD1a, GD1b, and GQ1b) Botulism PCR stool: *Clostridium baratii* Toxin F DNA by real-time PCR: DETECTED, confirmed by culture identifying active toxin F

### Differential diagnoses for botulism

As described in the case, treatment includes prompt administration of botulinum antitoxin, which neutralizes the circulating neurotoxin. Empiric therapy can prevent progression of paralysis and is most effective if used within 24 h of symptom onset, according to some literature (9). However, there was a review of the registry of patients receiving an equine-derived heptavalent botulinum antitoxin product for treatment of botulism neurotoxin serotypes A–G that indicated empiric treatment within 2 days of symptom onset was associated with decreased time in the ICU, decreased need for mechanical ventilation, and fewer days admitted to the hospital overall (10). In the case described above, we saw improvement even after administration more than 48 h from symptom onset, which is remarkable according to other data reviewed for this report, and one hypothesis could be serotype F leading to a milder form of botulism. Unfortunately, the exact dose and manufacturer of antitoxin this patient received were unavailable in the documentation of the health record.

It is important to utilize appropriate consult services for patients with suspected botulism, including infectious disease, who may help facilitate contact with the proper public health authorities to obtain antitoxin as well as investigate for possible outbreaks. Additional treatment measures include a high level of supportive care, typically in an intensive care setting. Per the CDC guidelines, standard isolation precautions should be used for patients with suspected and confirmed botulism. Antibiotics such as metronidazole can be used cautiously to clear the bacteria in the gastrointestinal tract, but priority should be focused on empiric antitoxin treatment. Once the patient is stabilized, an effort to maintain gut health to restore normal microbiota should be made (9).

### Conclusion

Empiric antitoxin therapy is a lifesaving measure in suspected *C. baratii* infections (9). The nonspecific nature of early symptoms necessitates a high degree of clinical vigilance and preparedness. Early intervention not only improves outcomes but also underscores the need for greater awareness of *C. baratii* botulism among healthcare professionals (9). The case we present demonstrates the effectiveness of empiric antitoxin. The patient was already intubated and on mechanical ventilation and even received tracheostomy; however, he was able to be decannulated during his admission and, after months of rehabilitation, made a full neurologic recovery. Future research should focus on improving diagnostic tools and understanding the epidemiology of *C. baratii* to further optimize treatment protocols.
